# Extracellular Myocardial Volume in Patients With Aortic Stenosis

**DOI:** 10.1016/j.jacc.2019.11.032

**Published:** 2020-01-28

**Authors:** Russell J. Everett, Thomas A. Treibel, Miho Fukui, Heesun Lee, Marzia Rigolli, Anvesha Singh, Petra Bijsterveld, Lionel Tastet, Tarique Al Musa, Laura Dobson, Calvin Chin, Gabriella Captur, Sang Yong Om, Stephanie Wiesemann, Vanessa M. Ferreira, Stefan K. Piechnik, Jeanette Schulz-Menger, Erik B. Schelbert, Marie-Annick Clavel, David E. Newby, Saul G. Myerson, Phillipe Pibarot, Sahmin Lee, João L. Cavalcante, Seung-Pyo Lee, Gerry P. McCann, John P. Greenwood, James C. Moon, Marc R. Dweck

**Affiliations:** a Centre for Cardiovascular Sciences, University of Edinburgh, Edinburgh, United Kingdom; b Barts Health NHS Trust and University College London, London, United Kingdom; c UPMC Cardiovascular Magnetic Resonance Center, Heart and Vascular Institute, Pittsburgh, Pennsylvania; d Department of Internal Medicine, Seoul National University Hospital, Seoul, Republic of Korea; e University of Oxford Centre for Clinical Magnetic Resonance Research, BHF Centre of Research Excellence (Oxford), NIHR Biomedical Research Centre (Oxford), Oxford, United Kingdom; f Department of Cardiovascular Sciences, University of Leicester and the NIHR Leicester Biomedical Research Centre, Glenfield Hospital, Leicester, United Kingdom; g Multidisciplinary Cardiovascular Research Centre & The Division of Biomedical Imaging, Leeds Institute for Cardiovascular and Metabolic Medicine, University of Leeds, Leeds, United Kingdom; h Institut Universitaire de Cardiologie et de Pneumologie de Québec/Québec Heart and Lung Institute, Université Laval, Québec City, Québec, Canada; i National Heart Center Singapore, Singapore; j Inherited Heart Muscle Disease Clinic, Department of Cardiology, Royal Free Hospital, NHS Foundation Trust, London, United Kingdom; k Division of Cardiology, Asan Medical Center Heart Institute, University of Ulsan College of Medicine, Seoul, Republic of Korea; l Charité Campus Buch ECRC, Berlin, and Helios Clinics Cardiology Germany, DZHK partner site, Berlin, Germany

**Keywords:** aortic stenosis, cardiovascular magnetic resonance, diffuse myocardial fibrosis, T1 mapping, CI, confidence interval, CMR, cardiovascular magnetic resonance, ECV, extracellular volume, ECV%, extracellular volume fraction, HR, hazard ratio, iECV, indexed extracellular volume, LA, left atrial, LV, left ventricular, NYHA, New York Heart Association, STS-PROM, Society of Thoracic Surgeons Predicted Risk of Mortality

## Abstract

**Background:**

Myocardial fibrosis is a key mechanism of left ventricular decompensation in aortic stenosis and can be quantified using cardiovascular magnetic resonance (CMR) measures such as extracellular volume fraction (ECV%). Outcomes following aortic valve intervention may be linked to the presence and extent of myocardial fibrosis.

**Objectives:**

This study sought to determine associations between ECV% and markers of left ventricular decompensation and post-intervention clinical outcomes.

**Methods:**

Patients with severe aortic stenosis underwent CMR, including ECV% quantification using modified Look-Locker inversion recovery–based T1 mapping and late gadolinium enhancement before aortic valve intervention. A central core laboratory quantified CMR parameters.

**Results:**

Four-hundred forty patients (age 70 ± 10 years, 59% male) from 10 international centers underwent CMR a median of 15 days (IQR: 4 to 58 days) before aortic valve intervention. ECV% did not vary by scanner manufacturer, magnetic field strength, or T1 mapping sequence (all p > 0.20). ECV% correlated with markers of left ventricular decompensation including left ventricular mass, left atrial volume, New York Heart Association functional class III/IV, late gadolinium enhancement, and lower left ventricular ejection fraction (p < 0.05 for all), the latter 2 associations being independent of all other clinical variables (p = 0.035 and p < 0.001). After a median of 3.8 years (IQR: 2.8 to 4.6 years) of follow-up, 52 patients had died, 14 from adjudicated cardiovascular causes. A progressive increase in all-cause mortality was seen across tertiles of ECV% (17.3, 31.6, and 52.7 deaths per 1,000 patient-years; log-rank test; p = 0.009). Not only was ECV% associated with cardiovascular mortality (p = 0.003), but it was also independently associated with all-cause mortality following adjustment for age, sex, ejection fraction, and late gadolinium enhancement (hazard ratio per percent increase in ECV%: 1.10; 95% confidence interval [1.02 to 1.19]; p = 0.013).

**Conclusions:**

In patients with severe aortic stenosis scheduled for aortic valve intervention, an increased ECV% is a measure of left ventricular decompensation and a powerful independent predictor of mortality.

Aortic stenosis is a disease of both the valve and myocardium. Progressive myocardial remodeling and hypertrophy occur over time in response to sustained pressure overload, decreasing wall stress, and maintaining cardiac performance. However, if untreated, this hypertrophic response eventually decompensates, and patients transition to symptomatic heart failure and adverse events (1).

Myocardial fibrosis is a key pathological process driving left ventricular (LV) decompensation (2). Two distinct patterns of fibrosis are observed: focal replacement fibrosis and diffuse interstitial fibrosis (3). Both forms of fibrosis can be detected noninvasively using cardiovascular magnetic resonance (CMR): replacement fibrosis with the late gadolinium enhancement technique and diffuse interstitial fibrosis with newer T1 mapping approaches. Although replacement fibrosis appears irreversible, regression of diffuse fibrosis is observed following relief of pressure overload with aortic valve intervention (4, 5, 6). Robust assessment of diffuse fibrosis is therefore desirable to identify early LV decompensation at a stage when pathological myocardial changes are largely reversible and targeted early valve intervention may improve patient outcomes.

Several T1 mapping measures have been proposed to date to detect changes in diffuse myocardial fibrosis. Native T1 mapping produces a voxel-based map of the myocardium that estimates absolute myocardial T1 values (7). Extracellular volume (ECV)-based measures utilize extracellular gadolinium-based contrast agents to calculate the relative (extracellular volume fraction [ECV%]) or absolute (indexed extracellular volume [iECV]) ECV of the myocardium (8,9). Although each measure has been validated against histology (8, 9, 10, 11, 12, 13, 14, 15, 16), the optimal T1 mapping approach remains unclear and robust multicenter outcome data are lacking.

In the present study, we investigated CMR T1 mapping in a large international multicenter study of patients with severe aortic stenosis scheduled for aortic valve intervention. In particular, we investigated the association between ECV-based measures and clinical characteristics, markers of LV decompensation and post-intervention clinical outcomes.

## Methods

### Patient populations

Patients with American Heart Association/American College of Cardiology/European Society of Cardiology criteria (17,18) for severe aortic stenosis who were awaiting aortic valve intervention were recruited as part of multiple prospective observational cohorts from 10 centers across Europe, North America, and Asia: the United Kingdom (The British Society of Cardiovascular Magnetic Resonance Consortium: Edinburgh, Leeds, Leicester, London [Barts Heart Centre], and Oxford), Germany (Berlin), United States (Pittsburgh), Canada (Québec), and South Korea (Seoul) (Online Table 1). All patients underwent CMR with T1 mapping performed both before and following intravenous gadolinium contrast administration. Exclusion criteria were the presence of an implantable cardiac device, advanced renal dysfunction (estimated glomerular filtration rate <30 ml/min/1.73 m^2^), previous valve replacement, and presence of another coexistent myocardial pathology such as cardiac amyloidosis, hypertrophic cardiomyopathy, or myocarditis. The study was conducted according to the Declaration of Helsinki and approved by the relevant local research ethics committees. Written informed consent was obtained from all participants. All patients underwent comprehensive medical history and physical examination. Transthoracic echocardiography was performed according to international clinical guidelines and within accredited tertiary echocardiographic units. Particular focus was placed upon measurement of aortic stenosis severity, which was assessed on the basis of the peak velocity, mean gradient, and aortic valve area (17, 18, 19).

### Cardiovascular magnetic resonance

CMR was performed on a range of different scanners, T1 mapping pulse sequences, and field strengths (Online Table 1). Standard long-axis cine images were acquired as well as a short-axis cine stack of the left ventricle. Late gadolinium enhancement imaging with both a short-axis LV stack and standard long-axis views was performed 5 to 15 min following gadolinium contrast agent administration. T1 mapping data were acquired in a short-axis mid-ventricular view of the left ventricle both before and 10 to 20 min following gadolinium contrast agent administration.

### Image post-processing and analysis

CMR image analysis was performed by 2 operators (R.J.E., T.A.T.) within a core lab according to a standardized analysis protocol (Online Appendix) using cvi42 software (Circle Cardiovascular Imaging, Calgary, Canada). The operators were blinded to the outcome data. Patients with CMR features consistent with a diagnosis of an alternative myocardial pathology were excluded (n = 5). The short-axis stack was contoured to calculate left and right ventricular volumes, ejection fraction, and LV mass, which were indexed to body surface area (calculated using the Mosteller formula). LV trabeculations and papillary muscles were included in the myocardial mass and excluded from the cavity volumes as per Society for Cardiovascular Magnetic Resonance recommendations (20). Left atrial (LA) volume was calculated via the biplane area–length method and indexed to body surface area (21).

The presence of noninfarct (mid-wall) and infarct patterns of late gadolinium enhancement were recorded and quantitative analysis performed using the full-width-at-half-maximum technique (20), with the extent of late gadolinium enhancement expressed as a percentage of total LV mass. Areas of signal contamination by epicardial fat or blood pool were manually excluded. Patients with clear imaging features of alternative myocardial pathology (e.g., amyloidosis) were excluded from further analysis.

Core lab T1 mapping analysis was performed using a standardized pre-specified analysis protocol. Epicardial and endocardial contours were manually drawn in the midinferoseptum (segment 9 of the standard 17-segment model [22]) on scanner-generated, short-axis, native and post-contrast T1 maps at the mid-ventricular level. A 10% offset was applied to minimize the influence of signal from the adjacent blood pool and epicardial fat. A septal segment was chosen because improved reproducibility has previously been demonstrated using septal regions of interest compared with analysis of all mid-ventricular segments on short-axis images (23). Segments containing noninfarct late gadolinium enhancement were included in the T1 mapping analysis, whereas those with infarct late gadolinium enhancement were excluded according to Society for Cardiovascular Magnetic Resonance guidelines (24). Native T1, ECV%, and iECV were then calculated (Online Appendix). Interobserver reproducibility of each of the T1 mapping measures was determined from independent analysis of 15 randomly selected scans. ECV% and iECV were pre-specified as the predominant T1 mapping measures for comparison because of the potential advantages these measures offer when comparing values acquired at different magnetic field strengths (25) and with different modified Look-Locker inversion recovery–based T1 mapping sequences (10).

### Longitudinal follow-up and clinical events

The primary outcome measure was all-cause mortality. The secondary outcome measure was cardiovascular mortality, which was defined as death attributable to myocardial ischemia or infarction, heart failure, cardiac arrest (due to arrhythmia or unknown cause), or cerebrovascular accident. Outcome events were adjudicated by review of patient health records (including the U.K. Spine database), and cause of death was adjudicated by 3 observers (P.B., J.P.G., M.R.D.). Among the centers in the United Kingdom, death certificates were available in all patients. Deaths occurring at international sites outside of the United Kingdom were adjudicated using a combination of medical record review, reports from family members, and death certificates where available.

### Statistical analysis

The distribution of all continuous variables was assessed using the Shapiro-Wilk test and presented using mean ± SD or median (interquartile range). Comparisons between groups were performed using the independent 2-sample Student's *t*-test or Mann-Whitney *U* test as appropriate. We presented all categorical variables as counts and percentages and used the Fisher exact test or chi-square test for comparison. The relationship between 2 continuous variables was assessed using Pearson’s r and Spearman’s rho as appropriate. Comparisons between ECV% and iECV tertiles were performed with 1-way analysis of variance or Kruskal-Wallis test as appropriate.

The influence of imaging center, CMR scanner manufacturer, magnetic field strength, and pulse sequence on T1 mapping values was analyzed using independent 2-sample Student's *t*-tests and linear regression analysis. Interobserver variability for native T1 and ECV% was determined by calculating the intraclass correlation coefficient on a random sample of 15 scans. Univariable linear regression was also performed to determine associations between clinical and imaging variables with T1 mapping measures. Multivariable linear regression was then performed using variables significantly associated with T1 measures as well as important variables (e.g., age and sex) regardless of strength of univariable association. Univariable Cox-regression analysis was performed to determine which variables were associated with all-cause mortality as the primary outcome measure as well as with cardiovascular mortality. Time to event or final status check was taken from the date of valve intervention. For all-cause mortality, variables with a significant association were included in the multivariable Cox regression model. The hazard ratio (HR) per unit increase in the variable of interest and 95% confidence intervals (CIs) were expressed as HR (95% CI). All statistical analyses were performed using SPSS version 24 (SPSS, IBM, Chicago, Illinois) and GraphPad Prism version 8.0 (GraphPad Software, San Diego, California). A 2-sided p < 0.05 was considered statistically significant.

## Results

**Figure 1:**
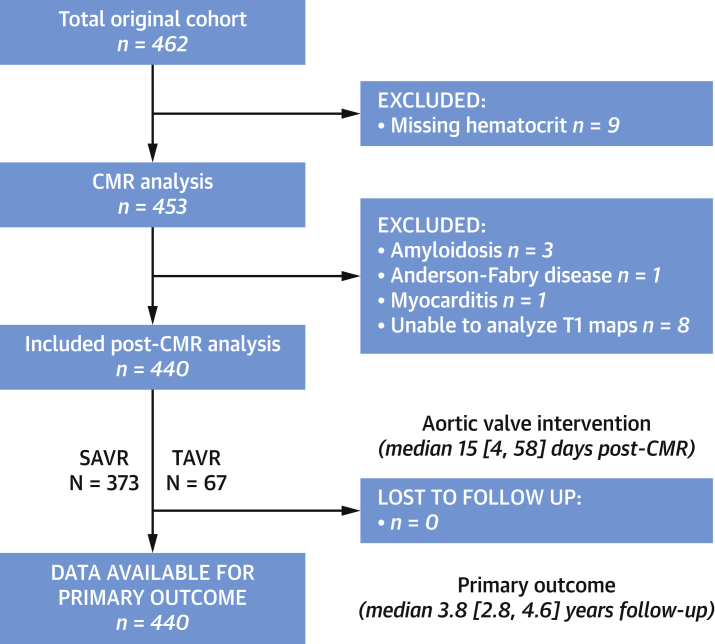
Flow Diagram of Study Participants CMR = cardiovascular magnetic resonance; SAVR = surgical aortic valve replacement; TAVR = transcatheter aortic valve replacement.

A total of 440 patients across 10 sites in 5 countries were included in the final analysis (70 ± 10 years, 59% male) (Figure 1) with a large proportion having hypertension (64%), diabetes mellitus (21%), and coronary heart disease (38%) (Table 1 and Online Table 2). Overall, 277 (63%) patients were imaged on 1.5-T and 163 (37%) patients on 3-T magnetic resonance scanners. Aortic valve intervention was performed at a median of 15 (IQR: 4 to 58) days following CMR. This was either isolated surgical aortic valve replacement (n = 311, 71%), combined coronary artery bypass grafting with surgical aortic valve replacement (n = 62, 14%), or transcatheter aortic valve replacement (n = 67, 15%).

**Table 1:** Baseline Characteristics and Imaging Results by ECV% Tertile

	ECV%			
	Tertile 1: <25.9% (n = 147)	Tertile 2: 25.9%–29.1% (n = 146)	Tertile 3: >29.1% (n = 147)	p Value∗
Age, yrs	68 ± 9	70 ± 10	71 ± 11	0.07
Male	84 (57)	89 (61)	86 (59)	0.80
Body mass index, kg/m^2^	27.9 ± 5.0	27.8 ± 5.5	27.1 ± 4.6	0.41
Body surface area, m^2^	1.86 ± 0.25	1.85 ± 0.23	1.84 ± 0.24	0.69
Past medical history				
Hypertension	90 (61)	93 (64)	97 (67)	0.60
Diabetes mellitus	25 (17)	29 (20)	39 (27)	0.11
Atrial fibrillation	15 (10)	15 (10)	26 (18)	0.09
Previous myocardial infarction	4 (3)	13 (10)	21 (16)	**0.002**
Coronary artery disease†	45 (12)	51 (35)	72 (49)	**0.003**
Clinical factors				
NYHA functional class III or IV	35 (27)	54 (43)	68 (55)	**<0.001**
Systolic blood pressure, mm Hg	131 ± 18	133 ± 22	128 ± 19	0.17
Diastolic blood pressure, mm Hg	73 ± 12	72 ± 11	73 ± 13	0.87
STS-PROM score, %	1.44 (0.88–2.29)	1.40 (0.92–2.15)	1.89 (1.13–3.31)	**<0.001**
EuroSCORE II, %	1.24 (0.82–2.19)	1.44 (0.99–2.21)	2.18 (1.14–4.28)	**<0.001**
Echocardiographic measures				
Peak aortic-jet velocity, m/s	4.48 ± 0.66	4.54 ± 0.80	4.35 ± 0.91	0.11
Peak aortic valve gradient, mm Hg	82 ± 24	85 ± 31	79 ± 33	0.20
Mean aortic valve gradient, mm Hg	50 ± 16	51 ± 19	48 ± 21	0.33
Aortic valve area, cm^2^	0.73 ± 0.19	0.76 ± 0.30	0.71 ± 0.25	0.20
Indexed aortic valve area, cm^2^/m^2^	0.40 ± 0.11	0.41 ± 0.15	0.39 ± 0.13	0.49
Valvuloarterial impedance, mm Hg/ml/m^2^	3.91 ± 1.17	3.85 ± 1.17	4.00 ± 1.02	0.52
Bicuspid aortic valve	47 (34)	47 (35)	50 (37)	0.87
Discordant echocardiographic measures of severity	25 (17)	25 (17)	33 (22)	0.40
Low-flow, low-gradient subtype (preserved or reduced ejection fraction)	7 (5)	6 (4)	13 (9)	0.16
Cardiovascular magnetic resonance				
Indexed left ventricular end-diastolic volume, ml/m^2^	70 ± 22	80 ± 29	85 ± 31	**<0.001**
Indexed left ventricular end-systolic volume, ml/m^2^	17 (11–28)	21 (14–36)	30 (17–51)	**<0.001**
Indexed left ventricular stroke volume, ml/m^2^	49 ± 12	51 ± 15	47 ± 13	**0.032**
Left ventricular ejection fraction, %	72 ± 13	67 ± 15	59 ± 18	**<0.001**
Left ventricular ejection fraction <50%	10 (7)	22 (15)	39 (27)	**<0.001**
Left ventricular mass index, g/m^2^	86 ± 28	94 ± 32	100 ± 35	**0.001**
Maximum left ventricular wall thickness, mm	15 ± 3	15 ± 3	15 ± 3	0.45
Mass/volume, g/ml	1.27 ± 0.35	1.25 ± 0.46	1.23 ± 0.36	0.68
Indexed right ventricular end-diastolic volume	64 ± 18	64 ± 16	67 ± 20	0.22
Indexed right ventricular end-systolic volume, ml/m^2^	21 (16–27)	21 (15–29)	23 (16–30)	0.18
Indexed right ventricular stroke volume, ml/m^2^	41 ± 11	42 ± 11	41 ± 10	0.77
Right ventricular ejection fraction, %	65 ± 9	65 ± 10	62 ± 13	**0.03**
Indexed left atrial volume, ml/m^2^	48 ± 21	54 ± 22	58 ± 25	**<0.001**
Late gadolinium enhancement	55 (37)	73 (50)	92 (63)	**<0.001**
Late gadolinium enhancement as a percentage of myocardial mass (full-width-at-half-maximum method), %	2.94 (1.61–4.26)	3.77 (1.89–7.48)	5.10 (2.36–7.93)	0.067
Late gadolinium enhancement (mid-wall pattern) present in segment 9	4 (3)	4 (3)	8 (5)	0.36
Hematocrit, %	0.41 ± 0.04	0.39 ± 0.04	0.38 ± 0.05	**<0.001**
Lambda	0.41 ± 0.04	0.45 ± 0.03	0.51 ± 0.05	**<0.001**
ECV%, %	23.9 ± 1.6	27.4 ± 1.0	31.7 ± 2.4	-
iECV, ml/m^2^	18.5 (15.3–22.4)	22.9 (18.9–28.9)	28.3 (22.4–35.1)	**<0.001**
Clinical events				
All-cause mortality, rate/1,000 patient-yrs	17.3	31.6	52.7	**0.009**
Cardiovascular mortality, rate/1,000 patient-yrs	4.0	5.7	18.6	**0.047**

Values are mean ± SD, n (%), or median (interquartile range), unless otherwise indicated. The p values in **bold** are statistically significant.

ECV% = extracellular volume fraction; iECV = indexed extracellular volume; NYHA = New York Heart Association; STS-PROM = Society of Thoracic Surgeons Predicted Risk of Mortality.

∗ The p values refer to tests for trends.

† Coronary artery disease defined as history of previous myocardial infarction, obstructive disease on angiography (stenosis >50% left main stem or >70% proximal epicardial coronary, artery) or previous coronary intervention.

### Native T1 values

**Figure 2:**
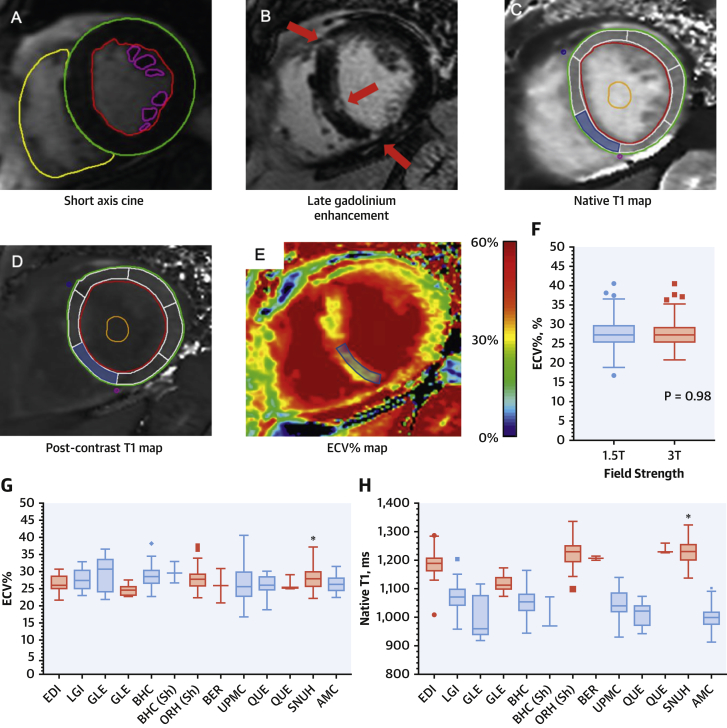
Multiparametric CMR Assessment Cardiovascular magnetic resonance (CMR) short-axis cine images were contoured to provide ventricular volumes, mass, and ejection fraction **(A)**. Areas of late gadolinium enhancement **(B, red arrows)** were quantified using the full-width-at-half-maximum technique. Native **(C)** and post-contrast **(D)** T1 maps were analyzed, and the mean value from segment 9 **(shaded blue)** and blood pool **(orange contour)** were used to calculate the extracellular volume fraction (ECV%). ECV% values did not vary by field strength (p = 0.98) **(F)**, and minimal variation in ECV% values was observed across the different centers **(G).** By contrast, native T1 values varied significantly by center **(H)**, mainly due to the effect of magnetic field strength (**blue** = 1.5-T, **red** = 3.0-T). Contour legend: **red** = left ventricular endocardial; **green** = left ventricular epicardial; **yellow** = right ventricular endocardial; **purple** = papillary muscle; **orange** = blood pool region of interest; **blue** = myocardial (segment 9) region of interest. AMC = Asan Medical Center, Seoul, Korea; BER = Berlin, Germany; BHC = Barts Heart Centre, London, United Kingdom; EDI = Edinburgh, United Kingdom; GLE = Leicester, United Kingdom; LGI = Leeds, United Kingdom; ORH = Oxford, United Kingdom; QUE = Québec, Canada; Sh = ShMOLLI T1 mapping sequence used; SNUH = Seoul National University Hospital, Seoul, Korea; UPMC = Pittsburgh, Pennsylvania.

In keeping with previous work (25,26), substantial variation in native T1 values was observed between the different centers (Figure 2). In particular, native T1 values were 20% higher in patients imaged at 3.0-T compared with 1.5-T (1,213 ± 57 ms vs. 1,042 ± 50 ms; p < 0.001). In an exploratory analysis, we adjusted native T1 values for local, sex-specific normal T1 values acquired on the same scanner using multiple methods (Online Methods, Online Table 3). Although these adjusted native T1 measurements correlated with markers of LV decompensation, they did not demonstrate an association with clinical outcomes (Online Results, Online Tables 4 to 7).

### ECV-based assessments (ECV% and iECV)

ECV% values were consistent across the different centers (Figure 2), with no differences between ECV% values in patients imaged at 1.5-T and 3.0-T (27.7 ± 3.7% vs. 27.7 ± 3.5%; p = 0.975). On univariable linear regression analysis, there was no association between ECV% values and either magnetic field strength (p = 0.975), scanner manufacturer (p = 0.416), or the T1 mapping sequence used (p = 0.246).

**Figure 3:**
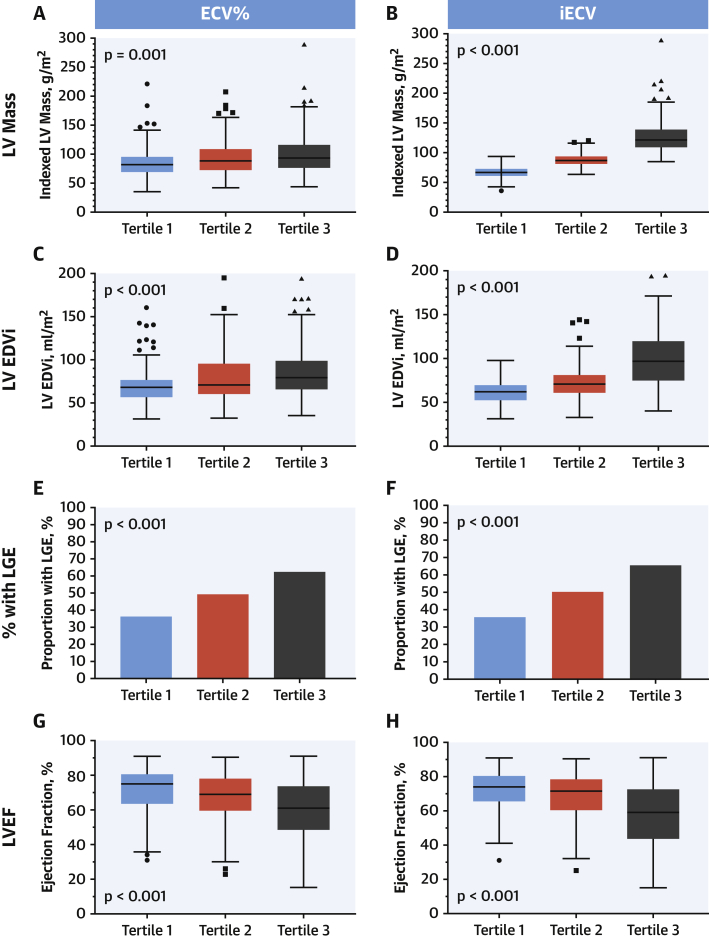
Markers of LV Decompensation Across ECV% and iECV Tertiles When comparing clinical and imaging variables across extracellular volume fraction (ECV%) tertiles, there was a progressive increase in LV mass **(A)**, LV end-diastolic volume **(C)**, and proportion of patients with late gadolinium enhancement **(E)**, with a reduction in LVEF **(G)**. A similar pattern was seen when comparing these characteristics across tertiles of indexed extracellular volume (iECV) **(B, D, F, and H)**. EDVi = indexed end-diastolic volume; LGE = late gadolinium enhancement; LV = left ventricle; LVEF = left ventricular ejection fraction.

The mean ECV% was 27.7 ± 3.6%, with good interobserver variability (4.4 ± 3.4%, intraclass correlation coefficient = 0.961). To explore associations between ECV% and clinical variables, the total cohort was divided into tertiles (tertile 1, <25.9%; tertile 2, 25.9 to 29.1%; tertile 3, >29.1%) (Table 1). Across the tertiles, there was a progressive increase in patients with established coronary heart disease (p = 0.003), Society of Thoracic Surgeons Predicted Risk of Mortality (STS-PROM), and EuroSCORE II risk scores (both p < 0.001). There was also progressive evidence of LV decompensation with more patients classified as New York Heart Association (NYHA) functional status III or IV (p < 0.001), more patients demonstrating late gadolinium enhancement (p < 0.001) and a progressive increase in indexed LA volumes, indexed LV volumes, and LV mass index across the tertiles (all p ≤ 0.001) (Table 1, Figure 3). Moreover, there was a fall in both left and right ventricular ejection fractions across the tertiles, albeit largely within the normal range (both p < 0.05) (Table 1). These analysis of variance associations remained present on the univariable analysis (Online Table 8), but on multivariable analysis, only increasing age (p = 0.028), LV ejection fraction (p < 0.001), and late gadolinium enhancement (p = 0.035) remained independently associated with ECV%.

Median iECV was 22.5 (18.1 to 29.6) ml/m^2^. Analysis by iECV tertile (tertile 1, <19.5 ml/m^2^; tertile 2, 19.5 to 26.9 ml/m^2^; tertile 3, >26.9 ml/m^2^) (Online Table 9) demonstrated a progressive increase in the proportion of males, subjects with coronary heart disease, and surgical risk scores (EuroSCORE II; p < 0.01 for all). Similar to ECV%, imaging markers of LV decompensation (LV mass, LV volumes, LA volumes, late gadolinium enhancement, and deterioration in LV and right ventricular ejection fractions) also progressed across the tertiles (Figure 3). Associations with iECV on univariable analysis were similar to the tertiles analysis (Online Table 10). On multivariable analysis, clinical measures independently associated with iECV were age, male sex, coronary heart disease, peak aortic-jet velocity, indexed LA volume, late gadolinium enhancement, and LV ejection fraction (p < 0.05 for all).

### Clinical outcomes

Clinical outcome data were collected from 440 patients after a median of 3.8 (IQR: 2.8 to 4.6) years. Final status checks were performed between January and August 2018, and no patient was lost to follow-up. Over this time, 52 deaths were observed (12%), of which 7 occurred within 30 days of valve intervention (1 perioperative death). Robust cause of death data was available in 37 of these events (71%), of which 14 (38%) were classified as a cardiovascular death following adjudication.

**Figure 4:**
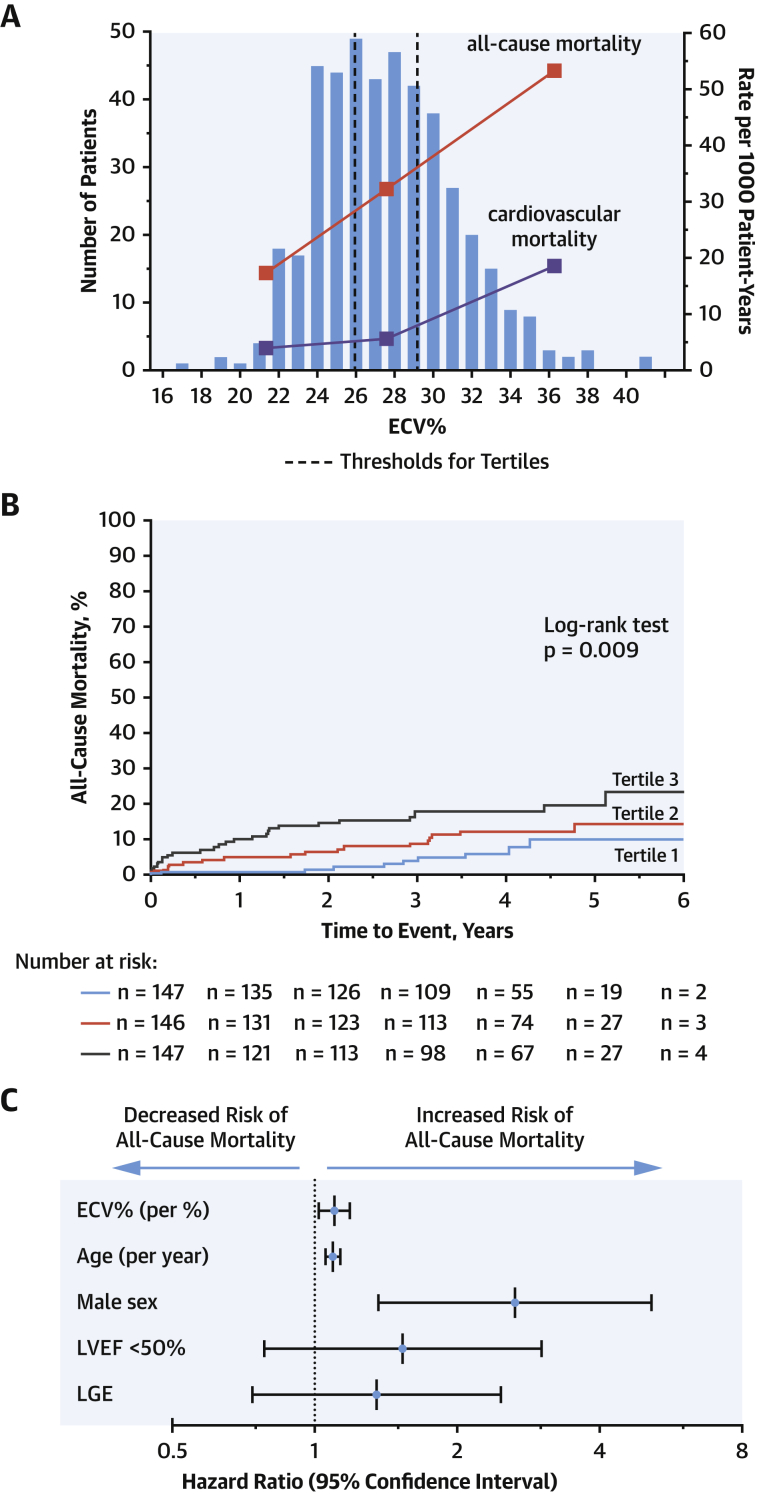
Distribution of ECV% and Relationship With Clinical Events ECV% is normally distributed **(A)**. When divided into tertiles, both the all-cause mortality rate **(red squares)** and cardiovascular mortality **(purple squares)** progressively increased across the tertiles. On Kaplan-Meier analysis, there was a progressive increase in all-cause mortality across tertiles of ECV% **(B)** (p = 0.009). ECV% remained an independent predictor of all-cause mortality on multivariable analysis **(C)** (hazard ratio: 1.10; p = 0.013). Abbreviations as in Figure 3.

All-cause mortality progressively increased across the ECV% tertiles, being approximately 3 times higher in the top versus the bottom tertile (tertile 1, 17.3 deaths; tertile 2, 31.6 deaths; tertile 3, 52.7 deaths per 1,000 patient-years; log-rank test p = 0.009) (Figure 4). This relationship appeared numerically consistent across intervention subgroups (Online Table 11) although the absolute number of events in these subgroups was small. Univariable Cox regression analysis showed a positive association between ECV% and mortality (HR: 1.15; 95% CI: 1.07 to 1.23; p < 0.001); other univariate predictors included age, male sex, STS-PROM score, EuroSCORE II, atrial fibrillation, indexed LA volume, coronary heart disease, and late gadolinium enhancement (all p < 0.05) (Table 2). ECV% was also associated with confirmed cardiovascular death on univariable analysis (HR: 1.22; 95% CI: 1.07 to 1.38; p = 0.003), as was late gadolinium enhancement (p = 0.012) (Online Table 12). ECV% remained associated with cardiovascular death when events that could not be classified due to insufficient information were also included in this endpoint (HR: 1.15; 95% CI: 1.05 to 1.26; p = 0.003).

**Table 2:** Univariable Cox Regression Analysis for All-Cause Mortality

	Univariable Analysis	
	Hazard Ratio (95% CI)	p Value
Age, yrs	1.09 (1.05–1.13)	**<0.001**
Male	2.45 (1.29–4.68)	**0.006**
STS-PROM score, %	1.37 (1.22–1.54)	**<0.001**
EuroSCORE II, %	1.15 (1.10–1.21)	**<0.001**
Known coronary disease	2.32 (1.34–4.00)	**0.003**
NYHA functional class III or IV	3.03 (1.63–5.61)	**<0.001**
Atrial fibrillation	3.41 (1.87–6.22)	**<0.001**
Peak aortic-jet velocity, m/s	0.68 (0.48–0.96)	**0.030**
Mean aortic valve gradient, mm Hg	0.98 (0.96–0.99)	**0.009**
Indexed aortic valve area, cm^2^/m^2^	0.76 (0.09–6.20)	0.80
Bicuspid aortic valve	0.57 (0.30–1.09)	0.088
LV ejection fraction <50%	1.85 (1.00–3.42)	**0.049**
Indexed LV end-diastolic volume, ml/m^2^	1.00 (1.00–1.01)	0.60
Indexed LV stroke volume, ml/m^2^	0.98 (0.95–1.00)	**0.035**
Indexed LV mass, g/m^2^	1.00 (0.99–1.01)	0.86
Indexed left atrial volume, ml/m^2^	1.02 (1.01–1.03)	**<0.001**
Valvuloarterial impedance	1.20 (0.96–1.49)	0.11
Presence of late gadolinium enhancement	1.84 (1.05–3.23)	**0.035**
Late gadolinium enhancement as a percentage of myocardial mass (full-width-at-half-maximum method), %	1.01 (1.00–1.02)	**0.009**
Right ventricular ejection fraction, %	0.97 (0.95–1.00)	**0.031**
Hematocrit, %	0.95 (0.90–1.01)	0.073
Lambda	1.06 (1.02–1.11)	**0.006**
ECV%, %	1.15 (1.07–1.23)	**<0.001**
iECV, ml/m^2^	1.02 (1.00–1.04)	0.120

The p values in **bold** are statistically significant.

CI = confidence interval; ECV = extracellular volume; LV = left ventricular; other abbreviations as in Table 1.

Inclusion of variables in the multivariable models was limited to prevent overfitting. In the first model, ECV% remained predictive of the primary outcome independent of age and sex (p = 0.003) (Table 3). In the second model, ECV% remained predictive independent of age, sex, LV ejection fraction <50%, and late gadolinium enhancement (HR: 1.10; 95% CI: 1.02 to 1.19; p = 0.013) (Figure 4). This association remained when peak aortic-jet velocity was added to model 2 (model 3: p = 0.033). Finally, ECV% was associated with outcome independent of STS-PROM risk score in the fourth model (HR: 1.09; 95% CI: 1.01 to 1.17; p = 0.027) and presence of coronary disease, advanced NYHA functional status, presence of atrial fibrillation, LV mass index, and LA volume index in the fifth model (HR: 1.09; CI: 1.00 to 1.19; p = 0.042).

**Table 3:** Multivariable Cox Regression Analysis of Association Between ECV% and All-Cause Mortality

	ECV%			
			95% CI for HR	
	p Value	HR	Lower	Upper
All-cause mortality				
Univariable				
Model 1				
ECV%	**<0.001**	1.145	1.068	1.228
ECV%	**0.003**	1.124	1.047	1.207
Age, yrs	<0.001	1.086	1.049	1.125
Male	0.001	2.921	1.520	5.614
Model 2				
ECV%	**0.013**	1.100	1.020	1.186
Age, yrs	<0.001	1.093	1.054	1.133
Male	0.004	2.649	1.363	5.148
LVEF <50%	0.213	1.535	0.782	3.012
Late gadolinium enhancement	0.329	1.351	0.738	2.475
Model 3				
ECV%	**0.033**	1.088	1.007	1.176
Age, yrs	<0.001	1.094	1.054	1.135
Male	0.005	2.591	1.325	5.067
LVEF <50%	0.233	1.527	0.761	3.064
Late gadolinium enhancement	0.508	1.233	0.663	2.293
Peak aortic-jet velocity, m/s	0.213	0.788	0.541	1.147
Model 4				
ECV%	**0.027**	1.087	1.009	1.171
STS-PROM score, %	<0.001	1.280	1.125	1.457
Model 5				
ECV%	**0.042**	1.091	1.003	1.187
Known coronary disease	0.028	1.965	1.077	3.585
NYHA functional class III/IV	0.024	2.102	1.103	4.007
Atrial fibrillation	0.013	2.602	1.223	5.538
LV mass index	0.313	0.994	0.983	1.006
LA volume index	0.204	1.007	0.996	1.018
Cardiovascular mortality				
Univariable				
ECV%	0.003	1.215	1.068	1.382

Late gadolinium enhancement incorporates both infarct and noninfarct patterns. p Values in **bold** are statistically significant.

HR = hazard ratio; LA = left atrial; LVEF = left ventricular ejection fraction; other abbreviations as in Tables 1 and 2.

There was no difference between all-cause mortality rates when the cohort was analyzed by iECV tertile (p = 0.72) (Online Table 9) nor was iECV associated with all-cause mortality or cardiovascular death using univariable Cox regression analysis (p = 0.12 and p = 0.32, respectively).

## Discussion

In patients with severe aortic stenosis undergoing aortic valve replacement, diffuse myocardial fibrosis quantified by CMR T1 mapping is an independent predictor of all-cause mortality (Central Illustration). These data show that both the percentage (ECV%) and total volume (iECV) of diffuse fibrosis associate with clinical and imaging measures of LV decompensation. ECV% provides the most powerful independent prognostic information, outperforming conventional markers including late gadolinium enhancement and ejection fraction, with a 1% rise in ECV% resulting in a 10% increase in mortality hazard. ECV-based T1 mapping indices, therefore, hold major promise as fully quantitative markers of myocardial fibrosis and LV decompensation in aortic stenosis.

**Central Illustration:**
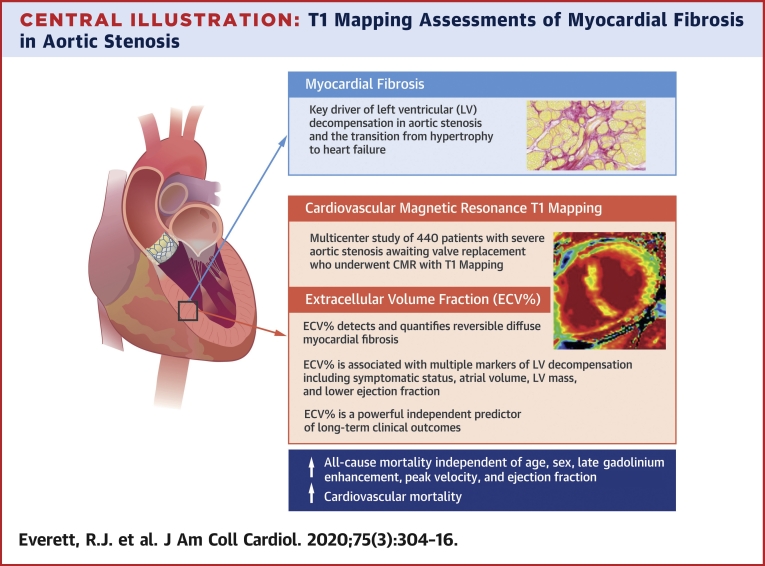
T1 Mapping Assessments of Myocardial Fibrosis in Aortic Stenosis Extracellular volume fraction (ECV%) using cardiovascular magnetic resonance (CMR) serves as an objective marker of left ventricular decompensation and is independently associated with long-term clinical outcomes in patients with aortic stenosis.

The 2 ECV-based measures examined in this study provide complementary information regarding diffuse myocardial fibrosis. ECV% provides a surrogate marker of the percentage of myocardium made up by fibrosis and has been validated extensively against histological fibrosis (8,10, 11, 12). Whereas ECV% offers a point assessment of fibrosis, serial ECV% measurements are insensitive to changes in fibrosis content, if fibrosis and cell volumes alter in proportion (6). By contrast, the iECV (ECV% × indexed LV myocardial volume) provides a surrogate measure of the absolute fibrosis burden (9) that can better track changes in fibrosis over time and in response to intervention such as valve replacement (6). In our cohort, both greater ECV% and iECV were associated with multiple features of a decompensating ventricle: advanced NYHA functional class and higher surgical risk scores, as well as higher LV volumes, LA volumes, presence and amount of late gadolinium enhancement, and worsening LV ejection fraction. Our data, therefore, support the utility of both ECV% and iECV as objective markers of LV decompensation in aortic stenosis. The lack of association of iECV with clinical outcomes likely reflects the study population, which also included a small proportion with discordant imaging measures, low-flow phenotypes, and a substantial female cohort who would be expected to have lower LV mass (driving a lower iECV measurement), but worse prognosis (27, 28, 29).

In aortic stenosis, prognostic T1 mapping data have been limited to single-center studies (9,30). This international multicenter investigation is the largest study to date to our knowledge, and the first to show a strong association between ECV% and all-cause mortality in patients with aortic stenosis on both univariable and multivariable models. After correction for a variety of other well-established prognostic markers, for every 1% increase in ECV%, there was a 10% increase in risk of all-cause mortality. These data are consistent with multiple previous studies demonstrating the prognostic utility in aortic stenosis of the other CMR marker of myocardial fibrosis, late gadolinium enhancement (9,28, 29, 30, 31). Whereas late gadolinium enhancement was again associated with an adverse prognosis in this cohort, this association was lost when multivariable models including ECV% were performed, suggesting ECV% measurement overlaps with some of the adverse signal seen with late gadolinium enhancement but also provides incremental prognostic information. At biopsy, even small areas of late gadolinium enhancement have been associated with thousands of microscars, which could be the primary driver of prognosis and may be better detected using ECV% compared with late gadolinium enhancement techniques (3). Further studies are now required to assess whether CMR assessments of myocardial fibrosis can improve the detection of LV decompensation in aortic stenosis and optimize the timing of aortic valve replacement (EVoLVeD [Early Valve Replacement Guided by Biomarkers of LV Decompensation in Asymptomatic Patients With Severe AS]; NCT03094143).

Importantly, our data confirm the feasibility of conducting international multicenter T1 mapping studies and comparing ECV-based values acquired on different scanners. The calculation of ECV% adjusts myocardial T1 for blood pool T1 measurements made on the same scanner. In principle, this should help correct for between-scanner differences in measuring T1 and enable comparison between ECV% values (10,25). Here, we observed no difference in ECV% values between patients imaged on Siemens or Phillips platforms, at 1.5- or 3.0-T, or using different pulse sequences (shortened vs. standard modified Look-Locker inversion recovery). This observation should now encourage similar multicenter T1 mapping studies, investigating ECV% and iECV, in other cardiovascular conditions.

### Study limitations

Native T1 has potential advantages as a marker of diffuse myocardial fibrosis, being based upon a single measurement and avoiding the need for gadolinium-based contrast administration. However, in this pragmatic multicenter setting, native T1 was hampered by considerable variation in values on different scanners. Recent studies have demonstrated that this variability can, in part, be addressed using phantom testing (31). This was not addressed here (the phantoms were not industrially manufactured at the time of recruitment) and requires further investigation in patients with aortic stenosis. Instead, we performed an exploratory analysis correcting native T1 for normal T1 values acquired in a sample of healthy volunteers imaged with the same pulse sequence and scanner. Although these adjusted native T1 values also demonstrated associations with markers of LV decompensation, they did not provide prognostic information. Prospective studies with more robust methods for correcting native T1 may be more successful. Subclinical cardiac amyloid deposition was not excluded; this would have required routine myocardial biopsies or bone scintigraphy, which were not felt warranted given the uncertain clinical importance of this observation.

Although no effect of T1 mapping pulse sequence on ECV% was demonstrated, this result cannot be extrapolated to saturation recovery-based T1 mapping techniques, which were not examined and may produce a lower ECV% value compared with inversion recovery sequences (32). Finally, no data were available regarding hospital admissions for heart failure, although previous studies have demonstrated a close association between this endpoint and all-cause mortality (33).

## Conclusions

ECV-based T1 mapping measurements are associated with multiple measures of LV decompensation in aortic stenosis. ECV% is a strong independent predictor of death in patients after aortic valve replacement, with further work now required to determine how these measures can be used to optimize the timing of aortic valve intervention.

Perspectives **COMPETENCY IN MEDICAL KNOWLEDGE:** Increased ECV% measured by cardiovascular magnetic resonance is an objective marker of left ventricular decompensation in patients with aortic stenosis (AS) that predicts mortality.

**TRANSLATIONAL OUTLOOK:** Future research should explore whether changes in ECV% can be used to guide the timing of intervention for patients with AS.
