# Supplementary material for: Extracellular Myocardial Volume in Patients With Aortic Stenosis
Source: J Am Coll Cardiol. 2020 Jan 28;75(3):304–16. doi: 10.1016/j.jacc.2019.11.032 (PMC6985897; doi:10.1016/j.jacc.2019.11.032)
Supplement: Online Data [file mmc1.docx]

**Supplementary Material**

Myocardial Extracellular Volume in Patients with Aortic Stenosis Undergoing Valve Intervention:

A Multicenter T1 Mapping Study

(ECV440 AS T1 mapping paper)

**Overview:**

1. **Supplementary methods (native T1 analysis)**
2. **Supplementary results (native T1 analysis)**
3. **Supplementary Tables 1-12**
4. **ECV440 image analysis protocol**

**Supplementary Methods**

**Native T1 analysis**

Given the variation in native T1 across the different centers and different field strengths we employed the following strategies to adjust native T1 for normal T1 values acquired on local scanners in healthy volunteers which were provided by the participating centers: 1) Native T1 treated as a dichotomous variable, with an increased native T1 defined as a value greater than the mean + 2 standard deviations (SD) of the native T1 values obtained in healthy volunteers on the local centre scanner and pulse sequence, 2) native T1 normalized to the local mean T1 value in healthy volunteers (observed native T1 time – mean native T1_HV_) and 3) the use of local normal ranges to calculate a Z-score ((observed native T1 time – mean native T1_HV_) / SD native T1_HV_), where T1_HV_ is the T1 time in healthy volunteers. Sex-specific normal range of native T1 was used where this information was available locally for the relevant scanner and pulse sequence.

Associations of these adjusted T1 values with markers of LV decompensations and clinical outcomes (all-cause mortality and cardiovascular mortality) were analyzed using univariable linear regression.

**Supplementary Results**

The mean and standard deviation T1 values for reference populations of healthy volunteers were available in eight centers (N= 400, Supplementary Table 8).

Native T1 was raised (as defined above) in 107 patients (27%). Those with abnormal native T1 were more often male, with a higher proportion of diabetes mellitus, coronary disease and late gadolinium enhancement on CMR, LV end-diastolic volume, mass and wall thickness were greater, and EuroSCORE II score was higher (all P<0.05, Supplementary Table 9). Similar results were obtained when native T1 was analyzed using both the T1 normalization or Z-score approaches, with increased corrected native T1 associated with male sex, presence of diabetes mellitus, coronary disease and late gadolinium enhancement, increased surgical risk scores and LV mass, and with reduced LV and RV ejection fraction (all P<0.05, Supplementary Tables 10 and 11). In both multivariable models, presence of coronary disease, late gadolinium enhancement and a lower LV ejection fraction remained independently associated with these adjusted native T1 values (all P<0.05).

There was no association seen between any of the adjusted native T1 values (when used as a dichotomous variable, T1 normalized to mean values in healthy volunteers or using the calculated native T1 Z-score) with either all-cause mortality or cardiovascular mortality (Supplementary Table 12). Multivariable analysis was therefore not performed.

**Supplementary Table 1: Cardiovascular magnetic resonance technical details and T1 mapping results by study centre**

| **Site** | **Scanner** | **Pulse sequence (pre)** | **Pulse sequence (post)** | **Contrast agent, dose and timing** | **N** | **Mean native T1, ms** | **Mean ECV%** |
| --- | --- | --- | --- | --- | --- | --- | --- |
| Edinburgh Heart Centre, UK | Siemens  Verio  3T | MOLLI  3(3)-3(3)-5 | MOLLI  3(3)-3(3)-5 | Gadovist  0.1 mmol/kg  20 mins | 28 | 1184±52 | 26.4±2.6 |
| Leeds Teaching Hospitals NHS Trust, UK | Phillips Achieva 1.5T | MOLLI  5(3)-3 | MOLLI  4(1)-3(1)-2 | Dotarem  0.2 mmol/kg  15 mins | 24 | 1071±53 | 27.7±3.0 |
| Glenfield Hospital, Leicester, UK | Siemens  1.5T  Skyra  3T | MOLLI  3(3)-3(3)-5 | MOLLI  3(3)-3(3)-5 | Gadovist  0.15 mmol/kg  20 mins | 5  10 | 997±80  1118±31 | 26.2±4.0 |
| Barts Heart Centre, London, UK | Siemens  Avanto  1.5T | MOLLI  5(3)-3  ShMOLLI  5(1)-1(1)-1 | MOLLI  4(1)-3(1)-2  ShMOLLI  5(1)-1(1)-1 | Dotarem  0.10 mmol/kg  15 mins | 131  3 | 1053±45  1002±59 | 28.6±3.1 |
| John Radcliffe Hospital, Oxford, UK | Siemens  Trio  3T | ShMOLLI  5(1)-1(1)-1  (WIP561) | ShMOLLI  5(1)-1(1)-1  (WIP561) | Omniscan  0.03 mmol/kg  15 mins | 17 | 1208±65 | 27.4±2.9 |
|  |  | ShMOLLI  5(1)-1(1)-1  (WIP448C) | ShMOLLI  5(1)-1(1)-1  (WIP448C) | Dotarem  0.10 mmol/kg  15 mins | 26 | 1224±52 | 28.6±3.6 |
| Berlin Ultrahigh Field Facility, Charité Campus Buch, Berlin, Germany | Siemens  Verio  3T | MOLLI  3(3)-3(3)-5 | MOLLI  3(3)-3(3)-5 | Gadovist  0.20 mmol/kg  10 mins | 2 | 1207±11 | 25.9±7.1 |
| University of Pittsburgh Medical Centre, Pittsburgh, PA, U.S.A. | Siemens  Espree  1.5T | MOLLI  5(3)-2 | MOLLI  4(1)-3(1)-2 | Gadovist  0.20 mmol/kg  20 mins  Prohance  0.20 mmol/kg  20 mins | 33  33 | 1045±45 | 26.6±5.0 |
| Québec Heart and Lung Institute, Québec, Canada | Phillips  Achieva  1.5T  Ingenia  3T | MOLLI  5(3)-3  MOLLI  5(3)-3 | MOLLI  5(3)-3  MOLLI  4(1)-3(1)-2 | Gadovist  0.20 mmol/kg  15 mins | 9  2 | 1013±45  1238±19 | 26.1±3.1 |
| Seoul National University Hospital, Seoul, South Korea | Siemens  Trio  3T | MOLLI  3(3)-3(3)-5 | MOLLI  3(3)-3(3)-5 | Magnevist  0.20 mmol/kg  10 mins | 77 | 1232±47 | 28.3±3.6 |
| Asan Medical Center, Seoul, South Korea | Siemens Avanto  1.5T | MOLLI  3(3)-3(3)-5 | MOLLI  3(3)-3(3)-5 | Gadovist  0.10 mmol/kg  20 mins | 40 | 1000±39 | 26.3±2.3 |

**Supplementary Table 2: Baseline characteristics, echocardiography and cardiovascular magnetic resonance imaging results by centre**

| **Site** | **Age, yrs** | **Male gender, n (%)** | **Coronary disease, n (%)** | **STS-PROM score, %** | **Peak aortic-jet velocity, m/s** | **AVAi, cm^2^/m^2^** | **Reduced EF <50%, n (%)** | **Late gadolinium enhancement, n (%)** | **iECV, mL/m^2^** |
| --- | --- | --- | --- | --- | --- | --- | --- | --- | --- |
| Edinburgh Heart Centre, UK  N=28 | 67±8 | 23 (82) | 6 (21) | 1.40 [0.85, 2.28] | 4.67±0.93 | 0.43±0.12 | 8 (29) | 10 (36) | 16.7 [13.9, 21.2] |
| Leeds Teaching Hospitals NHS Trust, UK  N=24 | 71±12 | 9 (38) | 12 (50) | 2.17, [1.40, 3.27] | 4.92±0.52 | 0.31±0.08 | 5 (21) | 15 (63) | 22.0 [18.2, 27.5] |
| Glenfield Hospital, Leicester, UK  N=15 | 75±13 | 8 (53) | 1 (7) | 1.28 [0.79, 2.45] | 4.40±0.53 | 0.41±0.12 | 2 (13) | 5 (33) | 17.1 [13.7, 19.8] |
| Bart’s Heart Centre, London, UK  N=134 | 71±9 | 74 (55) | 52 (39) | 1.45 [1.00, 2.42] | 4.38±0.58 | 0.38±0.11 | 21 (16) | 78 (58) | 22.1 [17.8, 28.0] |
| John Radcliffe Hospital, Oxford, UK  N=43 | 71±9 | 30 (70) | 19 (44) | 0.87 [0.76, 1.19] | 4.56±0.74 | 0.38±0.11 | 2 (5) | 28 (67) | 24.5 [19.7, 28.2] |
| Berlin Ultrahigh Field Facility, Charité Campus Buch, Berlin, Germany  N=2 | 69±7 | 2 (100) | 0 (0) | 2.76 | 5.04±0.81 | 0.34±0.05 | 0 (0) | 2 (100) | 24.4 |
| University of Pittsburgh Medical Centre, Pittsburgh, PA, U.S.A.  N=66 | 69±12 | 38 (58) | 50 (76) | 2.12 [1.29, 3.91] | 3.96±0.86 | 0.39±0.14 | 15 (23) | 35 (53) | 22.9 [17.8, 31.5] |
| Québec Heart and Lung Institute, Québec, Canada  N=11 | 62±9 | 8 (73) | 1 (9) | 0.81 [0.69, 1.07] | 3.79±0.65 | 0.44±0.07 | 0 (0) | 6 (55) | 16.8 [14.1, 19.2] |
| Seoul National University Hospital, Seoul, South Korea  N=77 | 70±7 | 48 (62) | 18 (23) | 1.64 [1.14, 2.38] | 4.60±0.90 | 0.46±0.17 | 7 (9) | 30 (39) | 26.6 [21.6, 36.0] |
| Asan Medical Center, Seoul, South Korea  N=40 | 64±13 | 19 (48) | 9 (23) | 1.48 [0.88, 2.44] | 4.95±0.81 | 0.38±0.09 | 11 (28) | 12 (30) | 26.2 [20.1, 34.7] |

**Supplementary Table 3: Native T1 reference ranges by centre where available**

|  |  |  | **Native T1 reference - men** | | | **Native T1 reference - women** | | |
| --- | --- | --- | --- | --- | --- | --- | --- | --- |
| **Centre** | **N** | **Field strength (T)** | **N** | **Mean±SD** | **Age** | **N** | **Mean±SD** | **Age** |
| **Barts** | **131** | 1.5 | 49 | 1008±33 | 51±14 | 45 | 1043±37 | 49±15 |
| **Barts** | **3** | 1.5 ShMOLLI | 49 | 948±26 | 51±14 | 45 | 966±31 | 49±15 |
| **Berlin** | **2** | 3.0 | 30 | 1155±53 | - | - | - | - |
| **Edinburgh** | **28** | 3.0 | 26 | 1165±29 | 61±19 | 17 | 1173±32 | 56±21 |
| **Leicester** | **5** | 1.5 | - | - | - | - | - | - |
| **Leicester** | **10** | 3.0 | 15 | 1084±30 | 69±10 | 7 | 1110±37 | 65±7 |
| **Leeds** | **24** | 1.5 | - | - | - | - | - | - |
| **Oxford** | **17** | 3.0 ShMOLLI* | 8 | 1154±30 | 59±5 | 11 | 1188±27 | 61±4 |
| **Oxford** | **26** | 3.0 ShMOLLI$ | 8 | 1147±30 | 59±5 | 11 | 1181±27 | 61±4 |
| **Pittsburgh** | **66** | 1.5 | 10 | 977±31 | 41±17 | 13 | 1008±15 | 42±16 |
| **Québec** | **8** | 1.5 | - | - | - | - | - | - |
| **Québec** | **3** | 3.0 | - | - | - | - | - | - |
| **SNUH** | **77** | 3.0 | 16 | 1202±20 | 68±5 | 17 | 1200±44 | 68±4 |
| **AMC** | **40** | 1.5 | 20 | 1013±46 | 46±16 | 20 | 1010±46 | 58±10 |
| **Total** | **440** |  |  |  |  |  |  |  |

* ShMOLLI WIP561

$ ShMOLLI WIP448C

*Pulse sequence is MOLLI unless stated otherwise.*

*AMC; Asan Medical Center, SD; standard deviation, SNUH; Seoul National University Hospital, T; Tesla*

**Supplementary Table 4: Demographic, clinical and imaging measures in patients with normal versus abnormal native T1 values (as assessed using local normal range)**

| **Variable** | **Normal native T1**  **N=293** | **Raised native T1**  **N=107** | **P value** |
| --- | --- | --- | --- |
| Age, years | 69±10 | 71±10 | 0.25 |
| Male sex, n (%) | 162 (55) | 76 (71) | **0.005** |
| Body mass index, kg/m^2^ | 27.4±5.0 | 28.0±5.2 | 0.37 |
| Body surface area, m^2^ | 1.83±0.24 | 1.88±0.24 | 0.07 |
| **Past medical history** |  |  |  |
| Hypertension, n (%) | 192 (66) | 73 (69) | 0.59 |
| Diabetes mellitus, n (%) | 59 (20) | 32 (30) | **0.04** |
| Atrial fibrillation, n (%) | 37 (13) | 14 (13) | 0.90 |
| Known coronary artery disease*, n (%) | 96 (33) | 58 (54) | **<0.001** |
| **Clinical factors** |  |  |  |
| NYHA functional class III or IV, n (%) | 97 (38) | 37 (43) | 0.41 |
| Systolic blood pressure, mmHg | 130±20 | 132±19 | 0.40 |
| Diastolic blood pressure, mmHg | 73±12 | 74±11 | 0.38 |
| STS-PROM score, % | 1.46 [0.98, 2.39] | 1.80 [1.12, 2.76] | 0.07 |
| EuroSCORE II, % | 1.39 [0.95, 2.42] | 1.95 [1.00, 4.02] | **0.012** |
| **Echocardiographic measures** |  |  |  |
| Peak aortic-jet velocity, m/s | 4.47±0.74 | 4.40±0.96 | 0.40 |
| Mean aortic valve gradient, mmHg | 50±18 | 50±22 | 0.90 |
| Aortic valve area, cm^2^ | 0.74±0.26 | 0.74±0.24 | 0.99 |
| Indexed aortic valve area, cm^2^/m^2^ | 0.41±0.13 | 0.39±0.13 | 0.45 |
| Valvuloarterial impedance, mmHg/mL/m^2^ | 3.93±1.16 | 3.94±1.03 | 0.93 |
| Bicuspid aortic valve, n (%) | 101 (36) | 35 (39) | 0.63 |
| **Cardiovascular magnetic resonance** |  |  |  |
| Indexed left ventricular end-diastolic volume, mL/m^2^ | 75±27 | 86±28 | **0.001** |
| Indexed left ventricular stroke volume, mL/m^2^ | 49±13 | 49±14 | 0.87 |
| Left ventricular ejection fraction, % | 68±15 | 62±19 | **<0.001** |
| Left ventricular mass index, g/m^2^ | 89±30 | 111±35 | **<0.001** |
| Maximum left ventricular wall thickness, mm | 15±3 | 16±3 | **<0.001** |
| Mass/volume, g/mL | 1.24±0.36 | 1.37±0.48 | **0.004** |
| Indexed right ventricular end-diastolic volume | 64±18 | 66±17 | 0.46 |
| Indexed right ventricular stroke volume, mL/m^2^ | 41±11 | 40±11 | 0.67 |
| Right ventricular ejection fraction, % | 65±11 | 62±11 | **0.05** |
| Indexed left atrial volume, mL/m^2^ | 52±22 | 53±18 | 0.54 |
| Late gadolinium enhancement, n (%) | 130 (44) | 67 (63) | **0.001** |
| Late gadolinium enhancement as a percentage of myocardial mass (full-width-at-half-maximum method), % | 3.76 [1.98, 7.60] | 4.26 [1.62, 7.08] | 0.82 |

**Supplementary Table 5: Linear regression analysis of associations between normalized native T1 (observed native T1 – mean native T1 in healthy volunteers) and demographic, clinical and imaging measures**.

|  |  | **Analysis of associations with native T1** | | | |
| --- | --- | --- | --- | --- | --- |
|  |  | **Unstandardized coefficients** | | **Standardized coefficients** |  |
|  | **Univariable analysis** | **B** | **SE** | **Beta** | **P value** |
| **Clinical factors** | Age, per 10 years | 1.900 | 2.560 | 0.370 | 0.460 |
|  | Male sex | 19.649 | 5.031 | 0.192 | **0.001** |
|  | Hypertension | 5.407 | 5.356 | 0.051 | 0.313 |
|  | Diabetes mellitus | 15.678 | 5.949 | 0.131 | **0.009** |
|  | Atrial fibrillation | 5.118 | 7.541 | 0.034 | 0.498 |
|  | STS-PROM score | 4.999 | 1.561 | 0.166 | **0.001** |
|  | EuroSCORE II | 1.878 | 0.909 | 0.108 | **0.039** |
|  | Known coronary disease | 17.931 | 5.093 | 0.174 | **<0.001** |
|  | NYHA functional class III or IV | 10.146 | 5.583 | 0.098 | 0.070 |
|  | Peak aortic-jet velocity, m/s | -1.850 | 3.159 | -0.030 | 0.558 |
|  | Indexed aortic valve area, cm^2^/m^2^ | -27.839 | 19.216 | -0.073 | 0.148 |
|  | Left ventricular ejection fraction, % | -0.770 | 0.149 | -0.250 | **<0.001** |
|  | Left ventricular mass index, g/m^2^ | 0.474 | 0.073 | 0.311 | **<0.001** |
|  | Indexed left atrial volume, mL/m^2^ | 0.217 | 0.124 | 0.089 | 0.081 |
|  | Right ventricular ejection fraction, % | -0.577 | 0.235 | -0.123 | **0.014** |
|  | Presence of late gadolinium enhancement | 28.125 | 4.842 | 0.280 | **<0.001** |
|  | Late gadolinium enhancement as a percentage of myocardial mass (full-width-at-half-maximum method), % | 0.035 | 0.228 | 0.011 | 0.879 |
|  |  |  |  |  |  |
|  | **Multivariable analysis** |  |  |  |  |
| **Model 1** | Age, per 10 years | -0.012 | 0.299 | -0.002 | 0.969 |
|  | Male sex | 13.049 | 5.778 | 0.125 | **0.025** |
|  | Diabetes mellitus | 11.674 | 6.506 | 0.097 | 0.074 |
|  | Known coronary disease | 14.026 | 5.968 | 0.134 | **0.019** |
|  | NYHA functional class III or IV | 5.263 | 5.793 | 0.050 | 0.364 |
|  | Peak aortic-jet velocity, m/s | 2.636 | 3.768 | 0.040 | 0.485 |
|  | Left ventricular ejection fraction, % | -0.497 | 0.213 | -0.160 | **0.020** |
|  | Indexed left atrial volume, mL/m^2^ | -0.092 | 0.145 | -0.036 | 0.526 |
|  | Right ventricular ejection fraction, % | -0.146 | 0.312 | -0.031 | 0.641 |
|  | Presence of late gadolinium enhancement | 18.067 | 5.929 | 0.176 | **0.003** |

*EF; ejection fraction, LV; left ventricle, NYHA; New York Heart Association, STS-PROM; Society of Thoracic Surgeons Predicted Risk of Mortality*

**Supplementary Table 6: Linear regression analysis of associations between native T1 (corrected using Z-score) and demographic, clinical and imaging measures**.

|  |  | **Analysis of associations with native T1** | | | |
| --- | --- | --- | --- | --- | --- |
|  |  | **Unstandardized coefficients** | | **Standardized coefficients** |  |
|  | **Univariable analysis** | **B** | **SE** | **Beta** | **P value** |
| **Clinical factors** | Age, per 10 years | 0.070 | 0.100 | 0.370 | 0.456 |
|  | Male sex | 0.529 | 0.193 | 0.136 | **0.006** |
|  | Hypertension | 0.117 | 0.204 | 0.029 | 0.567 |
|  | Diabetes mellitus | 0.708 | 0.226 | 0.156 | **0.002** |
|  | Atrial fibrillation | 0.254 | 0.287 | 0.044 | 0.375 |
|  | STS-PROM score | 0.262 | 0.060 | 0.225 | **<0.001** |
|  | EuroSCORE II | 0.126 | 0.035 | 0.188 | **<0.001** |
|  | Known coronary disease | 0.645 | 0.194 | 0.164 | **0.001** |
|  | NYHA functional class III or IV | 0.424 | 0.204 | 0.112 | **0.039** |
|  | Peak aortic-jet velocity, m/s | -0.187 | 0.120 | -0.078 | 0.120 |
|  | Indexed aortic valve area, cm^2^/m^2^ | -0.936 | 0.733 | -0.065 | 0.202 |
|  | Left ventricular ejection fraction, % | -0.033 | 0.006 | -0.281 | **<0.001** |
|  | Left ventricular mass index, g/m^2^ | 0.019 | 0.003 | 0.323 | **<0.001** |
|  | Indexed left atrial volume, mL/m^2^ | 0.007 | 0.005 | 0.077 | 0.132 |
|  | Right ventricular ejection fraction, % | -0.028 | 0.009 | -0.155 | **0.002** |
|  | Presence of late gadolinium enhancement | 0.986 | 0.185 | 0.258 | **<0.001** |
|  | Late gadolinium enhancement as a percentage of myocardial mass (full-width-at-half-maximum method), % | -0.001 | 0.009 | -0.006 | 0.930 |
|  |  |  |  |  |  |
|  | **Multivariable analysis** |  |  |  |  |
| **Model 1** | Age, per 10 years | -0.010 | 0.110 | -0.030 | 0.959 |
|  | Male sex | 0.095 | 0.214 | 0.024 | 0.659 |
|  | Diabetes mellitus | 0.499 | 0.241 | 0.112 | **0.039** |
|  | Known coronary disease | 0.490 | 0.221 | 0.127 | **0.028** |
|  | NYHA functional class III or IV | 0.193 | 0.215 | 0.050 | 0.369 |
|  | Peak aortic-jet velocity, m/s | 0.020 | 0.140 | 0.008 | 0.884 |
|  | Left ventricular ejection fraction, % | -0.022 | 0.008 | -0.192 | **0.006** |
|  | Indexed left atrial volume, mL/m^2^ | -0.004 | 0.005 | -0.047 | 0.406 |
|  | Right ventricular ejection fraction, % | -0.011 | 0.012 | -0.062 | 0.356 |
|  | Presence of late gadolinium enhancement | 0.575 | 0.220 | 0.152 | **0.009** |

*EF; ejection fraction, LV; left ventricle, NYHA; New York Heart Association, STS-PROM; Society of Thoracic Surgeons Predicted Risk of Mortality*

**Supplementary Table 7: Cox regression analysis of association between native T1 and clinical outcomes**

| **All-cause mortality** | |  |  |  |  | **95% CI for HR** | |
| --- | --- | --- | --- | --- | --- | --- | --- |
|  | **Variable** | **β** | **SE** | **P value** | **HR** | **Lower** | **Upper** |
| **Univariable** | Raised native T1 | 0.344 | 0.331 | 0.299 | 1.411 | 0.737 | 2.700 |
| **Univariable** | Normalized Native T1 | 0.005 | 0.003 | 0.132 | 1.005 | 0.999 | 1.011 |
| **Univariable** | Native T1 (Z score) | 0.087 | 0.078 | 0.263 | 1.091 | 0.937 | 1.271 |
|  |  |  |  |  |  |  |  |
| **Cardiovascular mortality** | |  |  |  |  | **95% CI for HR** | |
|  | **Variable** | **β** | **SE** | **P value** | **HR** | **Lower** | **Upper** |
| **Univariable** | Raised native T1 | -0.164 | 0.652 | 0.801 | 0.848 | 0.236 | 3.047 |
| **Univariable** | Normalized Native T1 | 0.005 | 0.005 | 0.324 | 1.005 | 0.995 | 1.016 |
| **Univariable** | Native T1 (Z score) | 0.183 | 0.127 | 0.148 | 1.201 | 0.937 | 1.539 |

**Supplementary Table 8: Univariable and multivariable associations with ECV%**

|  |  | **Analysis of associations with ECV%** | | | |
| --- | --- | --- | --- | --- | --- |
|  |  | **Unstandardized coefficients** | | **Standardized coefficients** |  |
|  | **Univariable analysis** | **B** | **SE** | **Beta** | **P value** |
| **Scan factors** | Magnetic field strength (3 T v 1.5 T) | 0.011 | 0.357 | 0.002 | 0.975 |
|  | T1 mapping sequence (ShMOLLI v MOLLI) | 0.654 | 0.563 | 0.055 | 0.246 |
|  | Scanner vendor (Phillips v Siemens) | -0.519 | 0.637 | -0.039 | 0.416 |
|  |  |  |  |  |  |
| **Clinical factors** | Age, per 10 years | 0.480 | 0.170 | 1.34 | 0.005 |
|  | Male sex | -0.069 | 0.351 | -0.009 | 0.843 |
|  | Hypertension | 0.224 | 0.361 | 0.030 | 0.535 |
|  | Diabetes mellitus | 0.842 | 0.421 | 0.095 | 0.046 |
|  | Atrial fibrillation | 1.012 | 0.515 | 0.093 | 0.050 |
|  | STS-PROM score | 0.614 | 0.105 | 0.280 | <0.001 |
|  | EuroSCORE II | 0.372 | 0.061 | 0.290 | <0.001 |
|  | Known coronary disease | 0.937 | 0.352 | 0.126 | 0.008 |
|  | NYHA functional class III or IV | 1.545 | 0.367 | 0.212 | <0.001 |
|  | Peak aortic-jet velocity, m/s | -0.529 | 0.216 | -0.117 | 0.015 |
|  | Indexed aortic valve area, cm^2^/m^2^ | 0.715 | 1.343 | 0.026 | 0.594 |
|  | Left ventricular ejection fraction, % | -0.072 | 0.010 | -0.322 | <0.001 |
|  | Left ventricular mass index, g/m^2^ | 0.023 | 0.005 | 0.207 | <0.001 |
|  | Indexed left atrial volume, mL/m^2^ | 0.030 | 0.008 | 0.188 | <0.001 |
|  | Right ventricular ejection fraction, % | -0.033 | 0.016 | -0.099 | 0.040 |
|  | Presence of late gadolinium enhancement | 1.645 | 0.336 | 0.228 | <0.001 |
|  |  |  |  |  |  |
|  | **Multivariable analysis** |  |  |  |  |
| **Model 1** | Age, per 10 years | 0.430 | 0.190 | 1.200 | 0.028 |
|  | Male sex | -0.461 | 0.392 | -0.063 | 0.240 |
|  | Diabetes mellitus | 0.140 | 0.433 | 0.016 | 0.746 |
|  | Atrial fibrillation | 0.220 | 0.601 | 0.021 | 0.714 |
|  | Known coronary disease | 0.480 | 0.385 | 0.066 | 0.213 |
|  | NYHA functional class III or IV | 0.566 | 0.383 | 0.078 | 0.140 |
|  | Peak aortic-jet velocity, m/s | -0.282 | 0.252 | -0.061 | 0.264 |
|  | Left ventricular ejection fraction, % | -0.062 | 0.014 | -0.281 | <0.001 |
|  | Left ventricular mass index, g/m^2^ | 0.008 | 0.008 | 0.062 | 0.307 |
|  | Indexed left atrial volume, mL/m^2^ | 0.009 | 0.010 | 0.059 | 0.334 |
|  | Right ventricular ejection fraction, % | 0.017 | 0.020 | 0.052 | 0.399 |
|  | Presence of late gadolinium enhancement | 0.805 | 0.381 | 0.112 | 0.035 |
|  |  |  |  |  |  |

*EF; ejection fraction, LV; left ventricle, NYHA; New York Heart Association, STS-PROM; Society of Thoracic Surgeons Predicted Risk of Mortality*

**Supplementary Table 9: Baseline characteristics and imaging results by iECV tertile**

|  | **Tertile 1**  **(<19.5 mL/m^2^)**  **N=147** | **Tertile 2**  **(19.5-26.9 mL/m^2^)**  **N=146** | **Tertile 3**  **(>26.9 mL/m^2^)**  **N=147** | **P value** |
| --- | --- | --- | --- | --- |
| Age, years | 70±10 | 70±10 | 69±11 | 0.714 |
| Male sex, n (%) | 60 (41) | 91 (62) | 108 (74) | **<0.001** |
| Body mass index, kg/m^2^ | 28.3±5.2 | 28.0±4.6 | 26.3±5.2 | **<0.001** |
| Body surface area, m^2^ | 1.84±0.23 | 1.87±0.24 | 1.82±0.25 | 0.193 |
| **Past medical history** |  |  |  |  |
| Hypertension, n (%) | 100 (68) | 92 (63) | 88 (61) | 0.418 |
| Diabetes mellitus, n (%) | 26 (18) | 33 (23) | 34 (23) | 0.433 |
| Atrial fibrillation, n (%) | 17 (12) | 17 (12) | 22 (15) | 0.608 |
| Previous myocardial infarction, n (%) | 10 (7) | 11 (8) | 17 (12) | 0.310 |
| Known coronary artery disease*, n (%) | 48 (33) | 52 (36) | 68 (46) | **0.041** |
| **Clinical factors** |  |  |  |  |
| NYHA functional class III or IV, n (%) | 49 (36) | 50 (40) | 58 (50) | 0.071 |
| Systolic blood pressure, mmHg | 134±18 | 131±20 | 126±20 | **0.004** |
| Diastolic blood pressure, mmHg | 74±12 | 73±13 | 70±12 | **0.008** |
| STS-PROM score, % | 1.36 [0.99, 2.26] | 1.57 [1.10, 2.48] | 1.72 [0.93, 2.61] | 0.149 |
| EuroSCORE II, % | 1.21 [0.94, 2.00] | 1.75 [0.98, 3.73] | 2.27 [1.10, 4.05] | **<0.001** |
| **Echocardiographic measures** |  |  |  |  |
| Peak aortic-jet velocity, m/s | 4.30±0.62 | 4.52±0.69 | 4.56±1.02 | **0.014** |
| Peak aortic valve gradient, mmHg | 76±21 | 84±25 | 87±38 | **0.012** |
| Mean aortic valve gradient, mmHg | 45±14 | 50±16 | 53±24 | **0.002** |
| Aortic valve area, cm^2^ | 0.76±0.24 | 0.71±0.23 | 0.73±0.28 | 0.307 |
| Indexed aortic valve area, cm^2^/m^2^ | 0.41±0.12 | 0.38±0.12 | 0.40±0.15 | 0.175 |
| Valvuloarterial impedance, mmHg/mL/m^2^ | 4.23±1.16 | 3.88±1.00 | 3.66±1.12 | **<0.001** |
| Bicuspid aortic valve, n (%) | 45 (32) | 47 (35) | 52 (38) | 0.595 |
| Discordant echocardiographic measures of severity, n (%) | 34 (23) | 23 (16) | 26 (18) | 0.246 |
| Low-flow low-gradient subtype (preserved or reduced ejection fraction), n (%) | 12 (9) | 2 (1) | 12 (9) | **0.015** |
| **Cardiovascular magnetic resonance** |  |  |  |  |
| Indexed left ventricular end-diastolic volume, mL/m^2^ | 62±14 | 74±19 | 100±33 | **<0.001** |
| Indexed left ventricular end-systolic volume, mL/m^2^ | 17 [11, 23] | 23 [15, 36] | 44 [23, 72] | **<0.001** |
| Indexed left ventricular stroke volume, mL/m^2^ | 44±9 | 49±11 | 53±17 | **<0.001** |
| Left ventricular ejection fraction, % | 72±11 | 69±14 | 57±19 | **<0.001** |
| Left ventricular ejection fraction <50%, n (%) | 6 (4) | 14 (10) | 51 (35) | **<0.001** |
| Left ventricular mass index, g/m^2^ | 66±11 | 87±12 | 127±30 | **-** |
| Maximum left ventricular wall thickness, mm | 13±2 | 15±2 | 17±3 | **<0.001** |
| Mass/volume, g/mL | 1.11±0.28 | 1.25±0.33 | 1.39±0.49 | **<0.001** |
| Indexed right ventricular end-diastolic volume | 60±14 | 65±18 | 70±20 | **<0.001** |
| Indexed right ventricular end-systolic volume, mL/m^2^ | 18 [14, 25] | 21 [15, 27] | 23 [17, 33] | **0.001** |
| Indexed right ventricular stroke volume, mL/m^2^ | 39±9 | 42±11 | 42±12 | **0.030** |
| Right ventricular ejection fraction, % | 65±9 | 66±8 | 61±13 | **<0.001** |
| Indexed left atrial volume, mL/m^2^ | 46±19 | 54±28 | 61±19 | **<0.001** |
| Late gadolinium enhancement, n (%) | 49 (33) | 74 (51) | 97 (66) | **<0.001** |
| Late gadolinium enhancement as a percentage of myocardial mass (full-width-at-half-maximum method), % | 2.21 [1.21, 3.91] | 3.76 [2.21, 7.54] | 5.25 [2.31, 8.19] | **<0.001** |
| Extracellular volume fraction, % | 25.6±2.9 | 27.6±3.1 | 29.8±3.5 | **<0.001** |
| Indexed extracellular volume (iECV), mL/m^2^ | 16.46[14.1, 18.1] | 22.5 [20.8, 24.3] | 33.4 [29.6, 39.8] | - |
| **Clinical events** |  |  |  |  |
| All-cause mortality, rate / 1000 patient years | 28.2 | 36.4 | 36.2 | 0.72 |

*NYHA; New York Heart Association, STS-PROM; Society of Thoracic Surgeons Predicted Risk of Mortality*

*known coronary artery disease defined as history of previous myocardial infarction, obstructive disease on angiography (stenosis >50% left main stem or >70% proximal epicardial coronary artery) or previous coronary intervention

**Supplementary Table 10: Univariable and multivariable associations with iECV**

|  |  | **Analysis of associations with iECV** | | | |
| --- | --- | --- | --- | --- | --- |
|  |  | **Unstandardized coefficients** | | **Standardized coefficients** |  |
|  | **Univariable analysis** | **B** | **SE** | **Beta** | **P value** |
| **Clinical factors** | Age, per 10 years | -0.550 | 0.470 | -0.560 | 0.244 |
|  | Male sex | 5.729 | 0.929 | 0.283 | **<0.001** |
|  | Hypertension | -0.959 | 0.994 | -0.046 | 0.335 |
|  | Diabetes mellitus | 2.228 | 1.160 | 0.092 | 0.055 |
|  | Atrial fibrillation | 0.300 | 1.430 | 0.010 | 0.834 |
|  | STS-PROM score | 1.022 | 0.304 | 0.166 | **0.001** |
|  | EuroSCORE II | 0.880 | 0.174 | 0.244 | **<0.001** |
|  | Known coronary disease | 2.116 | 0.976 | 0.103 | **0.031** |
|  | NYHA functional class III or IV | 2.177 | 0.925 | 0.120 | **0.019** |
|  | Peak aortic-jet velocity, m/s | 1.583 | 0.595 | 0.127 | **0.008** |
|  | Indexed aortic valve area, cm^2^/m^2^ | 0.160 | 3.714 | 0.002 | 0.966 |
|  | Left ventricular ejection fraction, % | -0.228 | 0.027 | -0.369 | **<0.001** |
|  | Indexed left atrial volume, mL/m^2^ | 0.115 | 0.020 | 0.268 | **<0.001** |
|  | Right ventricular ejection fraction, % | -0.154 | 0.044 | -0.167 | **<0.001** |
|  | Presence of late gadolinium enhancement | 5.246 | 0.922 | 0.263 | **<0.001** |
|  | Late gadolinium enhancement as a percentage of myocardial mass (full-width-at-half-maximum method), % | 0.086 | 0.047 | 0.125 | 0.071 |
|  |  |  |  |  |  |
|  | **Multivariable analysis** |  |  |  |  |
| **Model 1** | Age, per 10 years | -0.930 | 0.400 | -1.080 | 0.021 |
|  | Male sex | 3.816 | 0.790 | 0.218 | **<0.001** |
|  | Diabetes mellitus | 0.502 | 0.914 | 0.069 | 0.116 |
|  | Known coronary disease | 2.198 | 0.808 | 0.125 | 0.007 |
|  | NYHA functional class III or IV | 0.395 | 0.810 | 0.023 | 0.626 |
|  | Peak aortic-jet velocity, m/s | 1.795 | 0.515 | 0.161 | **0.001** |
|  | Left ventricular ejection fraction, % | -0.187 | 0.029 | -0.354 | **<0.001** |
|  | Indexed left atrial volume, mL/m^2^ | 0.065 | 0.018 | 0.170 | **<0.001** |
|  | Right ventricular ejection fraction, % | 0.009 | 0.043 | 0.011 | 0.835 |
|  | Presence of late gadolinium enhancement | 2.253 | 0.801 | 0.120 | **0.005** |

*NYHA; New York Heart Association, STS-PROM; Society of Thoracic Surgeons Predicted Risk of Mortality*

**Supplementary Table 11: All-cause mortality by Extracellular Volume Fraction Tertile by Subgroup of Type of Valve Intervention**

| **Subgroup** | **ECV% tertile** | **All-cause mortality, n** | **All-cause mortality rate / 1000 patient years** | **P value (comparison between tertiles)** |
| --- | --- | --- | --- | --- |
| **SAVR**  **(n=311)** | 1 | 4 | 10 | 0.026 |
|  | 2 | 9 | 24 |  |
|  | 3 | 14 | 40 |  |
| **SAVR+CABG**  **(n=62)** | 1 | 2 | 32 | 0.193 |
|  | 2 | 2 | 25 |  |
|  | 3 | 6 | 92 |  |
| **TAVR**  **(n=67)** | 1 | 3 | 67 | 0.932 |
|  | 2 | 6 | 83 |  |
|  | 3 | 6 | 82 |  |

*CABG; coronary artery bypass grafting, ECV%; extracellular volume fraction, SAVR; surgical aortic valve replacement, TAVR; transcatheter aortic valve replacement*

**Supplementary Table 12: Univariable cox regression analysis for cardiovascular mortality**

|  | **Univariable analysis** | |
| --- | --- | --- |
| **Variable** | **Hazard ratio (95% CI)** | **P value** |
| **Age**, years | 1.10 (1.03 to 1.17) | **0.007** |
| **Male sex** | 1.27 (0.43 to 3.80) | 0.67 |
| **STS-PROM Score,** % | 1.44 (1.22 to 1.71) | **<0.001** |
| **EuroSCORE II, %** | 1.16 (1.07 to 1.26) | **<0.001** |
| **Known coronary disease** | 3.23 (1.09 to 9.74) | **0.034** |
| **NYHA functional class III or IV** | 3.08 (0.93 to 10.2) | **0.066** |
| **Atrial fibrillation** | 4.53 (1.51 to 13.6) | **0.007** |
| **Peak aortic-jet velocity,** m/s | 0.36 (0.18 to 0.72) | **0.004** |
| **Mean aortic valve gradient,** mmHg | 0.95 (0.91 to 0.98) | **0.005** |
| **Indexed aortic valve area,** cm^2^/m^2^ | 2.04 (0.04 to 94.0) | 0.72 |
| **Bicuspid aortic valve** | 0.77 (0.24 to 2.46) | 0.66 |
| **LV ejection fraction < 50%** | 0.84 (0.19 to 3.72) | 0.81 |
| **Indexed LV end-diastolic volume,** mL/m^2^ | 1.00 (0.98 to 1.02) | 0.99 |
| **Indexed LV stroke volume,** mL/m^2^ | 0.98 (0.94 to 1.03) | 0.39 |
| **Indexed LV mass,** g/m^2^ | 1.00 (0.98 to 1.02) | 0.84 |
| **Indexed left atrial volume,** mL/m^2^ | 1.02 (1.01 to 1.04) | **0.008** |
| **Valvuloarterial impedance** | 0.91 (0.56 to 1.49) | 0.72 |
| **Presence of late gadolinium enhancement** | 13.6 (1.78 to 104) | **0.012** |
| **Late gadolinium enhancement as a percentage of myocardial mass (full-width-at-half-maximum method), %** | 1.00 (0.97 to 1.04) | 0.915 |
| **Right ventricular ejection fraction, %** | 1.01 (0.95 to 1.07) | 0.83 |
| **ECV%,** % | 1.22 (1.07 to 1.38) | **0.003** |
| **iECV,** mL/m^2^ | 1.02 (0.98 to 1.07) | 0.321 |

*CI; confidence interval, ECV; extracellular volume, iECV; indexed extracellular volume, LV; left ventricular, NYHA; New York Heart Association, STS-PROM; Society of Thoracic Surgeons Predicted Risk of Mortality*

**ECV440: Pre-specified Image analysis protocol**

**(adapted from BSCMR valve consortium guidance for AS700 study, Musa, Treibel et al, Circulation 2018)**

1. **Left and right ventricular volume and mass quantification**
   1. **The left ventricle**
   2. **The right ventricle**
2. **Left atrial volume**
3. **Late gadolinium enhancement**
4. **T1 mapping**

**1. Left and right ventricular volume and mass quantification**

(adapted from Schulz-Menger et al. Journal of Cardiovascular Magnetic Resonance 2013, 15:35)

- For each study, LV and RV volumes and LV mass are to be contoured by the same one individual using the dedicated LV short axis cine stack.
- If no intra- or extracardiac shunts are present, the RV and LV stroke volumes should be nearly equal (small differences are seen as a result of bronchial artery supply). Since the LV stroke volume is more reliably determined than the RV stroke volume, the LV data can be used to validate RV data.
- Manual contouring performed in cvi42 using the Bezier tool is the suggested method of analysis; the fully automated contour detection option is to be avoided.

**1a. The left ventricle**

- The LV end-diastolic and end-systolic image should be chosen as the images with the largest and smallest LV blood volumes respectively (for their identification, the full image stack should be evaluated).
- Deviations may occur, and extra care should be taken in the setting of LV dyssynchrony or severe mitral regurgitation. Aortic valve closure defines end-systole.
- If a slice is uninterpretable (e.g. degraded by triggering/breathing artefact) it should be excluded from systolic and diastolic measurements of both the LV and RV.
- The LV outflow tract is included as part of the LV blood volume. When aortic valve cusps are identified on the basal slice(s) the contour is drawn to include the outflow tract to the level of the aortic valve cusps.
- Care must be taken with the one or two most basal slices. A slice that contains blood volume at end-diastole may include only left atrium (LA) without LV blood volume at end-systole. The LA can be identified when less than 50% of the blood volume is surrounded by myocardium and the blood volume cavity is seen to be expanding during systole.
- Papillary muscles are to be **EXCLUDED** from the LV cavity for the purpose of analysis and **INCLUDED** within the LV mass (thus do require specific delineation).
- Epicardial borders should be drawn on the middle of the chemical shift artefact line (when present).
- Absolute LV mass is derived from diastolic epicardial and endocardial delineation; systolic epicardial contours are NOT required.
- Maximal LV wall thickness is measured as the thickest portion of the interventricular septum in short axis at end diastole (mm)
- When the most basal slice contains only a small crescent of basal lateral myocardium and no discernible ventricular blood pool, an epicardial contour for the visible myocardium is included for LV mass only.
- Similarly, when the most apical slice contains only a circle of myocardium without cavity blood pool, an epicardial contour without an endocardial contour should be drawn for LV mass calculations

Left ventricular (LV) chamber quantification. For LV chamber quantification, the endocardial (blue) and epicardial (yellow) contours are delineated in diastole in a stack of short axis slices that cover the whole left ventricle. c) illustrates the approach with Exclusion of the papillary muscles as part of the LV volume.

**1b. The right ventricle**

- As for the LV, it may be necessary to review all image slices in the stack to define end-diastole and end-systole for the RV.
- Trabeculations of the RV should be ignored, and a smooth endocardial border drawn to improve reader reproducibility (RV trabeculae and papillary muscles are typically included in RV volumes).
- Again, if no intra- or extracardiac shunts are present, the RV and LV stroke volumes should be nearly equal (small differences are seen as a result of bronchial artery supply).
- Since the LV stroke volume is more reliably determined than the RV stroke volume, the LV data can be used to validate RV data.
- The pulmonary valve may be visualized, and contours are included just up to, but not superior to this level.

Right ventricular (RV) chamber quantification. For RV volume quantification, the endocardial (red) contours are delineated in diastole (top) and systole (bottom) or short-axis (c and d) slices that cover the whole RV.

**2. Left atrial volume quantification**

- Measurement of left atrial (LA) volume is by the biplane area–length method.
- Images are analysed in the viewer module of cvi42 with a dual panel display selected to permit synchronisation of HLA and VLA by phase.
- All measurements are taken from the two-chamber (A) and four-chamber (B) views at end-ventricular systole, ensuring maximal LA size.
- The atrial endocardial border is traced to determine LA area with exclusion of the pulmonary veins, LA appendage, and mitral valve recess.
- LA length is measured from the midpoint of the mitral annulus plane to the posterior aspect of the left atrium. Left atrial volume (LAV) was calculated using the formula:

LAV = 8 x (A2Ch) x (A4Ch) / 3πL

- where A2Ch and A4Ch refer to the LA area in the two-chamber and four-chamber views,

respectively, and L is the shorter of the two LA length measurements (L2Ch, L4Ch) from these views.


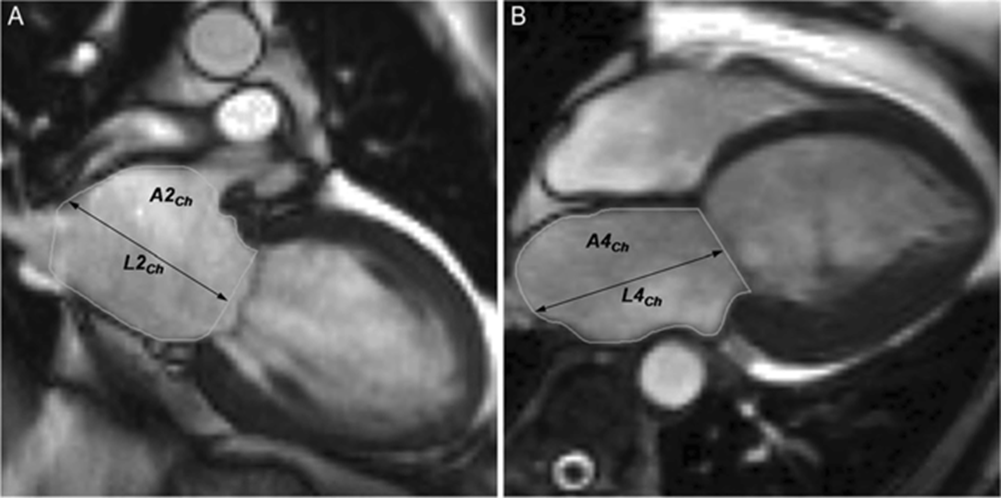


Gulati et al. 2013. European Journal of Heart Failure; 15(6); 660-670.

**3. Late gadolinium enhancement quantification**

- All images are to be quantified using CVI 42.
- The short axis LV stack acquired 10-15 minutes following gadolinium contrast administration is used for the purposes of late gadolinium quantification.
- Each slice is visually inspected by an experienced CMR reader for the presence or absence of gadolinium enhancement. Phase swap and other geometry images were used in order to assist in decision making where required.
- In only those slices deemed to have late gadolinium enhancement present, epi and endocardial contours should be manually drawn, with care take to exclude artefact, blood pool, fat and pericardium.
- The auto-identification tool is then applied, and an area of normal remote myocardium defined alongside identification of areas with increased signal intensity.
- Any hyperintense regions felt to be related to artefact are manually excluded.
- The 5SD technique should be used to determined late gadolinium enhancement mass.
- Late gadolinium enhancement mass is then divided by absolute LV mass as determined from the SA cine measurements to arrive at the % late gadolinium enhancement mass.

**4.** **T1 mapping analysis**

- All T1 mapping analysis should be performed using the T1 mapping module of CVI42
- The mid-ventricular short axis native T1 map should be loaded into the viewer (scanner generated motion-corrected T1 maps are preferred).
-
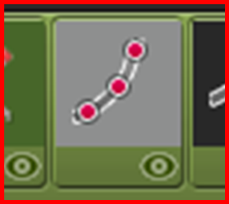
The endocardial and epicardial contours should be drawn using the Bezier tool (‘click-draw’ icon displayed).
- Anterior and posterior RV insertion points should be marked with the appropriate tool.
- The manual epicardial and epicardial offsets should both be set to “10%” and the number of segments per slice changed to “6”. This should result in automatic segmentation in the right-hand display into the 6 mid-ventricular myocardial segments (segments 7-12 of the AHA/ACC model).
- The blood pool contour should be drawn in the centre of visible blood pool with care taken to avoid papillary muscles within the region of interest.
- The above process should be repeated using the post-contrast T1 map at the same slice position (again, scanner-generated map is preferred). The blood pool contour should be copied from the native T1 map with adjustments applied as needed to avoid papillary muscles or artefact.
- The mean native and post-contrast T1 values for segment 9 are then recorded along with the mean blood pool native and post-contrast T1 values. If mid-wall late gadolinium enhancement is present in segment 9 this is included in the analysis, however if subendocardial infarct late gadolinium enhancement is present then this area is manually excluded (as per SCMR post-processing guidance). In the case of extensive infarction late gadolinium enhancement in segment 9, a separate unaffected mid-ventricular myocardial segment may be selected for T1 analysis.
- **Extracellular volume fraction (ECV%)** is then calculated using the following equations:
  - ECV% = partition coefficient x [1-hematocrit] x 100 *(to give a percentage value from 0-100)*
  - Partition coefficient = [∆R1myocardium / ∆R1blood-pool]
  - ∆R1 = (1 / post-contrast T1) - (1 / native T1)
- **Indexed extracellular volume (iECV)** is calculated using the following equation:
  - iECV = (ECV% x indexed LV mass) / (myocardial density x 100)
  - Myocardial density = 1.05 g/mL
